# SUMOhunt: Combining Spatial Staging between Lysine and SUMO with Random Forests to Predict SUMOylation

**DOI:** 10.1155/2013/671269

**Published:** 2013-06-17

**Authors:** Amna Ijaz

**Affiliations:** National Institute of Biotechnology and Genetic Engineering, P.O. Box 577, Jhang Road, Faisalabad, Pakistan

## Abstract

Modification with SUMO protein has many key roles in eukaryotic systems which renders the identification of its target proteins and sites of considerable importance. Information regarding the SUMOylation of a protein may tell us about its subcellular localization, function, and spatial orientation. This modification occurs at particular and not all lysine residues in a given protein. In competition with biochemical means of modified-site recognition, computational methods are strong contenders in the prediction of SUMOylation-undergoing sites on proteins. In this research, physicochemical properties of amino acids retrieved from AAIndex, especially those involved in docking of modifier and target proteins and optimal presentation of target lysine, in combination with sequence information and random forest-based classifier presented in WEKA have been used to develop a prediction model, SUMOhunt, with statistics significantly better than all previous predictors. In this model 97.56% accuracy, 100% sensitivity, 94% specificity, and 0.95 MCC have been achieved which shows that proposed amino acid properties have a significant role in SUMO attachment. SUMOhunt will hence bring great reliability and efficiency in SUMOylation prediction.

## 1. Introduction

Posttranslational modifications on proteins offer spectacular diversity and functional variety to an organism's otherwise constrained proteome. SUMOylation is one such PTM whose vast expanse of biological implications in organisms has brought it under attention; still till now many of its functional outcomes are not known. To name a few, SUMOylation is involved in transcriptional regulation [1–3], mRNA metabolism [4], apoptosis [5, 6], nuclear and subcellular transport [7, 8], protein trafficking [9], signal transduction [10], regulation of DNA damage and replication, cell-cycle progression, competition with other members of the ubiquitin family [2, 11, 12], prevention or promotion of deacetylation [13], chromosome segregation [14], structural integrity of chromatin and many proteins, and mitosis [15]. It has been reported to be involved in the perception of sound as well [16]. Also, it is known to participate in early developmental processes like cell differentiation, specification, division, and lineage commitment [17]. SUMOylation of a target protein can change its localization in a cell by altering its intermolecular and intramolecular interactions [18]. Hence, by determining whether a protein is SUMOylated or not, vital evidences can be gathered regarding its function and spatial association [19].

SUMO, a member of the ubiquitin family, is made up of 97 amino acids and is also called Smt3p, Pmt2p, PIC-1, GMP-1, Ubl1, and Sentrin [20]. It mostly modifies proteins present in the nucleus, cytoplasm, and sometimes the plasma membrane of eukaryotic cells [17]. SUMO proteins are highly conserved across eukaryotic proteomes. In mammals, including humans, there are four isoforms of SUMO called SUMO 1, SUMO 2, SUMO 3, and SUMO 4; in yeasts there is only one SUMO protein while plants produce at least eight SUMO isoforms [21]. SUMO 1, SUMO 2, and SUMO 3 are expressed throughout an organism's body [2] with the latter two having greater sequence similarity as compared to SUMO 1 [22]. SUMO 4 dominates in lymph node, kidney, and spleen in mammals [23], having resemblance with SUMO 2 and 3 and a dominant occurrence in kidney [24].

SUMOylation can occur in either the cytoplasm or the nucleus depending on the locality of the target protein, though the modification may afterwards be responsible for regulation of production and change in its localization [2] mostly from other parts of cell to the nucleus. RanGAP1, which controls the transportation of ribonucleoproteins across the nuclear pore complex, was identified as the first SUMO target [25]. Before SUMOylation, this protein mainly resides in the cytoplasm and afterwards associates itself with the cytoplasmic fibers of the nuclear pore complex [25]. All essential components of SUMOylation are present at the nuclear pore complex which shows its involvement in nuclear import or even retention of any incoming proteins within the nucleus [26, 27]. However, TEL protein, modified at K99, dominates in the cytoplasm while TEL mutated and hence unable to be SUMOylated at this residue mostly resides in the nucleus, pointing towards possible role of SUMOylation in its export [28].

Since SUMOylation is a part of a very wide array of biological and cellular processes, even a minor dysfunction in the pathway can result in severe pathological conditions like cancer. Neurodegenerative diseases including Huntington's disease [19], Alzheimer's disease, Parkinson's disease [29], and neuronal intranuclear inclusion disease [9] often have anomalies in the SUMOylation pathway involved in their onset. Also, type I diabetes [30] and familial amyotrophic sclerosis [13] have SUMOylation dysfunction as a frequent part of their beginning.

**Figure 1:**
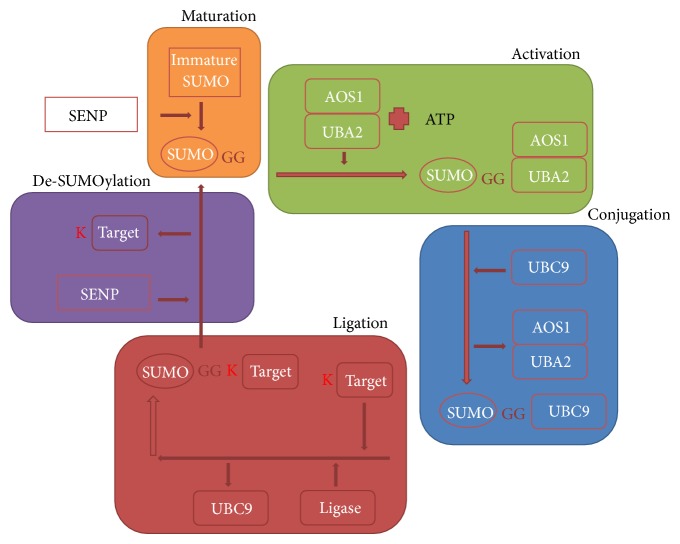
The SUMOylation pathway: the SUMO pathway is categorized into maturation, activation, conjugation, and ligation. First of all, SENPs convert immature SUMO to mature SUMO by exposing diglycine motif at carboxyl terminal. This mature SUMO is activated by AOS1/UBA2 (E1—activating enzyme) with the help of ATP-dependent reaction. Activated SUMO conjugates with E2—conjugating enzyme ubc9—which finally transfers it to target lysine on substrate protein with the help of E3 ligase.

SUMO proteins attach covalently to lysine, which mostly lies in consensus motif ΨKXE [31], where Ψ is any hydrophobic amino acid, K is the target lysine residue, X is any of the twenty encoded amino acids, and E is glutamic acid, with the help of SUMO E2-conjugation enzyme, ubc9 [31]. In mammals, any one of the four isoforms of SUMO conjugates to target protein as an individual molecule or in the form of polymeric chains [32] at target lysine. The enzymes working the SUMOylation pathway are E1-activating enzyme, E2-conjugating enzyme, and E3 ligase. The SUMO pathway can be categorized into maturation, activation, conjugation, and finally ligation at target site (Figure 1). First of all, SUMO is processed by SUMO-specific proteases (SENPs) [33] to cleave its terminal into an exposed diglycine motif at the carboxyl end, converting it from immature to mature protein. The maturation process of all three major SUMO proteins is identical in mammals [34]. Secondly, activation of mature SUMO takes place through an ATP-dependent thioester bond formation between SUMO and UBA2/AOS1, a heterodimeric E1-activating enzyme [2]. Direct linkage occurs between SUMO and UBA2 subunit of the heterodimer. This enzyme is called SAE1/SAE2 or Sua1/hUba2 in humans [34]. The yeast homologue of UBA2 subunit is Uba2p, which together with Aos1 was the first activating enzyme to be discovered [35] while the human homologue is hUba2 [36]; both act in the same way. The third step in this pathway is catalyzed by E2-conjugating enzyme, Ubc9. Activated SUMO is transferred from E1-activating enzyme to a cysteine residue in Ubc9. In contrast to other enzymes involved in the process, Ubc9 is the only type of enzyme identified in its category [31, 37]. It is Ubc9 which identifies the consensus or nonconsensus sequence at target site for subsequent conjugation [38]. In the final step, SUMO is attached to target protein with isopeptide bond between the exposed diglycine on carboxyl terminal of SUMO and the *ε*-amino group of target lysine in protein with the help of E3-ligating enzyme. Three distinct characteristics of ligating enzymes in SUMOylation pathway have been sketched through research: (i) they should be able to directly or indirectly associate with the target protein, (ii) they should be able to bind with their preceding enzyme, ubc9, in the pathway, and (iii) they should be capable of transferring SUMO to target protein or another SUMO in case of poly-SUMOylation [34]. RanBP2 which is a nuclear pore protein [39], TOPORS [40, 41], PIAS proteins [42], Pc2 [43] which is polycomb group protein, and RNF4 [44] have all been identified to have E3 ligase activity in SUMOylation pathway.

Nearly all research on SUMO attachment has pointed out the significance of ΨKXE motif, but on the other hand, SUMOylation has been reported to occur in regions outside of this consensus motif as well: in nonconsensus sites. Xu et al. [21] reported 26% SUMO occurrence in nonconsensus while Xue et al. [45] reported 23% such cases in bulk data used to develop their respective prediction servers. For example, there are four core histones, H2A, H2B, H3, and H4, that are frequently SUMOylated. However, none of the SUMOylated sites in these histones conform to the common consensus motif [46]. Several other consensus motifs have been proposed including NDSM and PDSM. NDSM proposes that negatively charged amino acids around the target lysine enhance SUMOylation [47] while PDSM is based on ΨKXEXXSP motif, both being only an extension of the original common motif [48].

An analysis of available PDB structures of several protein targets having 57 reported SUMOylation sites revealed 54 of the sites to be exposed on the surface while only 3 were buried within the proteins' globular structure [21]. Also, research has led to the conclusion that SUMOylation is greatly enhanced when the target lysine is forced to adopt a favorable conformation [49]. From this behavior it can be inferred that the conjugation enzymes and ligases have sequence preference since they come into direct contact with target protein. SUMOylation pathway requires only three enzymes, specificity of subcellular localization and appropriate presentation of target residue on globular structure [2]. In case of other PTMs, a variety of enzymes with their target recognition and modification systems bring out varied site preference; typically they are not focused on any one type of residue. The major role in SUMOylation is played by only few enzymes discussed above; it suffers from the lack of efficient target recognition and modification systems, thus emphasizing the importance of motif and sequence information on the target protein as a device of recognition in the pathway. 

Including sequence information as a principal contributor of computational prediction performance can provide rational computational tools, but focusing entirely on the consensus motif is not preferable as it can result in missing many true positives that lie in nonconsensus regions along with high false positive rate due to the many consensus sites that are actually not SUMOylated. In view of the occurrence of SUMO at both consensus and nonconsensus, sites it is proposed that other than the raw amino acid sequence around a SUMOylatable lysine residue there are factors of appropriate presentation and exposure of lysine and adjacent residues including steric hindrance, hydrophobicity, polarity, and entropy, playing a crucial role in determining whether a residue shall undergo SUMOylation or not. However, two residues downstream and one residue upstream of the target lysine play the most important role in SUMOylation [2, 50, 51].

We have employed different peptide lengths centered on lysine residues experimentally proved to undergo SUMOylation with sixteen amino acid properties (Table 1) from AAIndex to develop a prediction model named *SUMOhunt*. These amino acid properties were chosen on the basis of their contribution in increasing structural complementarity and association between incoming SUMO and target protein. Promising accuracy measures obtained on SUMOhunt developed by combining these properties with sequence information in the vicinity of target lysine and random-forest based algorithm presented in data mining software WEKA [52] have opened new paths for the development of an efficient prediction method. 

## 2. Materials and Methods

### 2.1. Dataset

452 modified lysine instances (positive instances) were obtained from dbPTM [53], from training sets and sites supplemented for SUMOpre [21] and SUMOsp [45] and in the publication titled *SUMO targets and Site Prediction: Combining Pattern Recognition and Phylogenetic Conservation* by Xue et al. [54]. Primary sequences around these residues were retrieved from UniprotKB [55] in the form of 21-mer peptides: SUMOylated lysine residue had 10 residues upstream and 10 residues downstream of it. Standard single letter code was used for every amino acid residue.

Removal of redundant information gave 293 modified residues within 181 proteins (S1). The remaining 7346 lysine residues in these proteins, that is, lysine residues not reported to be modified, were assumed to be unmodified (negative instances) from a total lysine count of 7639 as calculated from MAPRes [56], and 21-mer peptides were generated for them as well. Such cases that were reported as both modified and unmodified were considered modified.

It is better to use numerical data for the development of a predictor, so every amino acid was encoded furthermore with the coefficients given in numeric matrices of chosen amino acid properties (Table 1) in the AAIndex [57]. Each peptide could be represented by a 16∗21 dimension feature vector; that is, it could have 336 possible feature dimensions vectors.

This data was made workable with WEKA [52] by converting all the information into CSV format followed by conversion to ARFF. 

### 2.2. Training the Prediction Program

In WEKA [52], random forest algorithm was trained using the ARFF generated. With default settings, ten trees were trained to vote for the class of each given instance. The random forest was trained using a dataset that had equivalent amount of modified and unmodified instances; the unmodified instances were randomly selected from the larger bulk of 7346 sites.

The choice of 21-mer peptide length around the target lysine site was tested against the same sites being present in 11-mer and 7-mer peptides as well. Each peptide in the latter categories could be represented in 16∗11 = 176 and 16∗7 = 112 dimension feature vectors, respectively. Hence, three datasets made in exactly the same manner were obtained. The datasets were not “formally” divided into training or test sets for accuracy measurement; instead *percentage split *in WEKA [52] was employed for this purpose to optimize the size of train and test. The dictated percentage of the original data including the most *ideal-to-train *instances is extracted and used as training data while the rest is used to test (S2 and S3).

Accuracy measures were calculated using

(1) $$\begin{array}{c} \text{SN}=\frac{\text{TP}}{\text{TP}+\text{FN}},\\ \text{SP}=\frac{\text{TN}}{\text{TN}+\text{FP}},\\ \text{MCC}=\frac{\left(\text{TP}\times \text{TN}\right)-\left(\text{FP}\times \text{FN}\right)}{\sqrt{\left(\left(\text{TP}+\text{FP}\right)\left(\text{TP}+\text{FN}\right)\left(\text{TN}+\text{FP}\right)\left(\text{TN}+\text{FN}\right)\right)}}. \end{array}$$

### 2.3. Cross-Validation and Evaluation

**Figure 2:**
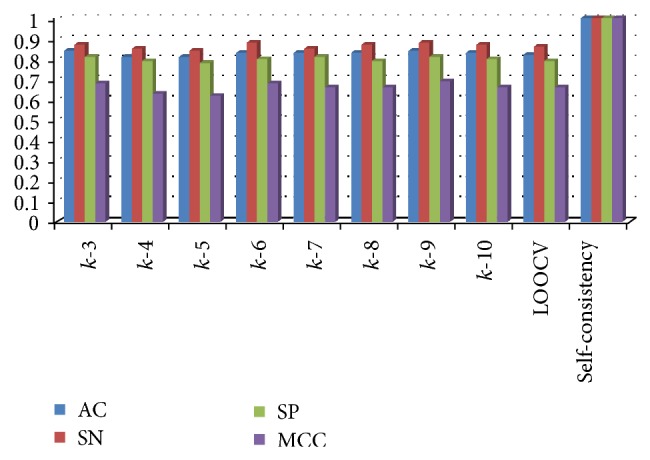
Comparison of accuracy measures (*y*-axis) at several *k*-fold cross-validations, LOOCV and self-consistency test (*x*-axis). In *k*-fold cross-validation, after dividing dataset into *k* sections, typical training procedure is conducted using *k* − 1 sections while the remaining one is used as test. This is repeated *k* times until every set has been used as test exactly once. For eight *k*-fold cross-validations performed, the value of *k* was kept from 3 to 10 having average AC at 81%, SN at 83%, SP at 79%, and MCC at 0.66. LOOCV is a type of *k*-fold cross-validation in which *k* is equal to the number of total instances. It has the similar average as *k*-fold. Self-consistency is a type of test in which prediction of every instance is done using the rules of the training dataset itself. This is done for each and every instance in the training dataset. In this test, 100% result is achieved for all accuracy measures.

**Figure 3:**
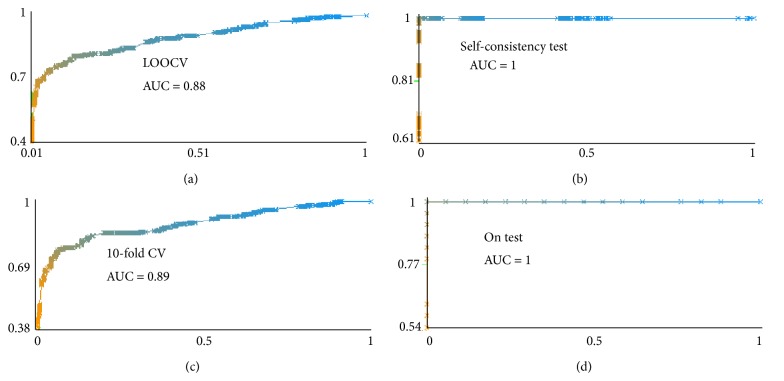
Area under the ROC (*y*-axis: true positive rate (fraction of true positives out of total positives) and *x*-axis: false positive rate (fraction of false positives out of total negatives)) of 10-fold cross-validation, self-consistency test, and LOOCV-ROC curve depicts the performance of a classifier by plotting true positive rate versus the false positive rate. The greater the area under the curve, higher is the performance of a classifier. For 10-fold cross-validation 89% area under the curve is obtained, for LOOCV it is 88%, while for self-consistency it is a full 100%. At the end, ROC for classification of test set has been done with an AUC of 1.

To test our predictor's power, we used the methods of Xu et al. [21], that is, self-consistency test, k-fold cross-validation, and jack knife (leave-one-out cross-validation). Specificity, sensitivity, accuracy, and correlation coefficient for these tests were computed (Figures 2 and 3).

*Self-consistency test*: it is predicted whether a given instance is positive or negative using the rules of the training dataset itself. This is done for each and every instance in the training dataset. 

*K-fold cross-validation*: here the dataset is randomly divided into *k* sections. Typical training procedure is conducted using *k* − 1 sections while the remaining one is used as test. This is repeated *k* times until every set has been used as test exactly once. 

*Jack knife cross-validation*: this is also called leave-one-out cross-validation and is an extension of the *k*-fold cross-validation, having *k* equal to the exact number of instances in the dataset.

### 2.4. Algorithm

The random forest algorithm [58] in WEKA [52] is built on decision tree classification. The said number of decision trees is generated with each tree having paths and nodes. Every node then uses rules derived from patterns in the data to decide between two or more paths. A given instance is classified by the last rule. To develop and grow the decision trees, a random selection of inputs and features is done at each node.

Voting on the class for a given instance is then carried out by the trees. Significant increase in classification accuracies have been observed if assortment of classifier trees is used and allowed to vote for the most popular class. Often random vectors are generated which govern the growth of each tree in the assortment. To see how a random forest actually shapes its model we should know that inherently for the *k*th tree a random vector Θ*k* is generated, independent of all the previous vectors produced, but it has the same distribution. Now a tree is grown using the training set provided and Θ*k*, which makes a classifier *h*(**x**, Θ*k*); here **x** is the input vector. The nature and dimension of Θ*k* depends on its use in the construction of the tree. After a large number of trees have been generated, they then vote for the most popular class.

It is difficult to interpret models developed through random forest [58]; but there are certain features which make it suitable for the prediction of PTMs: a mixture of discrete and continuous descriptors, binary, or multiclass data can be proficiently treated with random forest algorithm. This algorithm is successful even when there is a lot of disorder in the data [59].

## 3. Results

### 3.1. Frequency Analysis

**Figure 4:**
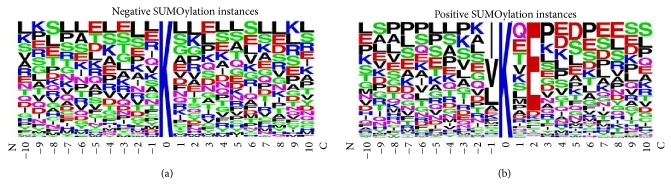
Frequency plot developed for all modified and unmodified instances with ten residues upstream and downstream of the target lysine; the plots are quite different from each other as expected. In the case of modified sites, there is a dominance of the conventional ΨKXE motif. Ψ is hydrophobic amino acid. Results show that aliphatic hydrophobic residues including valine, leucine, and isoleucine are dominant as compared to aromatic ones. K is the target lysine residue. X is any amino acid, which the results have shown it to be predominantly polar like glutamic acid, glutamine, and threonine. E is glutamic acid. In the dataset used here, about 76% sites follow the consensus motif.

The number of different types of amino acid residues prevalent around the modified sites was analyzed (Table 2) with all results substantiated through corresponding frequency plot (Figure 4) of the same dataset. Results confirm the prevalence of the ΨKXE motif; approximately only 24% modified sites in this research lack the consensus motif. At −1 position or in place of Ψ, it is not just any hydrophobic amino acid; the data based on 293 modified instances has a significantly higher occurrence of hydrophobic amino acids with aliphatic side chains: Val (73 occurrences), Leu (45), and Ile (92) as compared to those with aromatic side chains: Phe (9), Tyr (2), Trp (0), and His (1) and other hydrophobic residues. In place of X or at +1 position a dominance of polar amino acids including Glu (33), Gln (43), and Thr (32) is there. The bulky aromatic: Trp (3), Tyr (4), and His (4) and small-size amino acids: Asn (6) and Cys (3) are in a significantly lesser proportion. At +2 position, polar residues Glu and Asp are prevalent with 224 and 13 existences, respectively.

The incidence of Trp and Cys is strikingly low at all twenty positions around the target lysine, the highest being only 11 for Cys at position +10. The other three aromatic amino acids Phe, Tyr, and His also do not prevail around the target lysine, supporting the imperative role of a catalytically favorable presentation of lysine in its SUMOylation. Aromatic molecules could potentially compromise this presentation leading towards the unavailability of target lysine to incoming SUMO.

### 3.2. Optimum Window Length for Prediction

**Figure 5:**
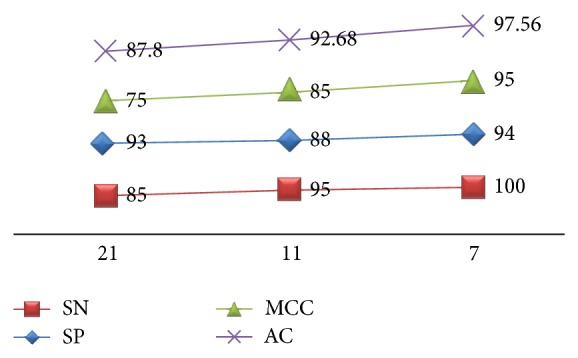
Comparison of accuracy measures at three window sizes: 21, 11, and 7 residue peptides; a good prediction model is dependent on optimum window size (*x*-axis) around target lysine. Window size 7 having 3 residues upstream and downstream is the best with highest AC, MCC, SP, and SN (*y*-axis) while window size 21 with 10 residues upstream and downstream has the lowest efficiency.

In order to derive a good prediction model, the optimum window length of the peptides used for its training has to be determined. As shown in Figure 5, values for all four accuracy measures are highest for peptides of window size 7 as compared to others. For 93% split (discussed in Section 2.2) of original dataset to train (7% is test) at window size 21 lowest accuracy of 87.8% is encountered in comparison to 92.7% at 11 window size and 97.56% at 7 window size. MCC and sensitivity show significant ascensions: from 75% for 21-mer peptide to 95% for 7-mer peptide and from 85% for 21-mer to 100% for 7-mer, respectively. Specificity does not show any major improvement or decrease among the three peptide lengths. Interestingly, an evaluation conducted for the same accuracy measures against window size during development of SUMOpre [21] also resulted in the same window size being chosen as the best length. Hence, with all the information and comparisons, window size 7, which has 3 residues downstream and 3 residues upstream of the target lysine residue, is the most rational choice for our prediction model.

### 3.3. Prediction Accuracy and Stability of Model

Prediction accuracy was measured using varied sequence lengths, with and without physicochemical properties. The addition of information on physicochemical properties of amino acid residues produced powerful accuracies given in Table 3. From the several models generated, the best with the chosen window size was further substantiated by testing its stability through three procedures discussed in Section 2.3. In Figure 2, accuracy measures for several *k*-fold cross-validations, jack-knife and self-consistency tests have been visualized. Results of the former two types of tests were all of nearly equal values with small deviations from mean while the latter varied significantly towards greater robustness; ROC for these tests can be observed in Figure 3. Average values of *k*-fold tests and LOOCV for AC remained at 82%, SN at 86%, SP at 80%, and MCC at 0.66. The last type of test, self-consistency, gave a 100% result as shown in Figure 2.

## 4. Discussion

The large range of biological processes and localizations populated by SUMO targets presents a great motivation to unravel information regarding SUMOylation and its targets by all possible means. Rigorous wet-lab experiments are frequently undertaken to isolate, identify, quantify, and report SUMOylation. SUMOylated proteins have been identified in yeast strains [51, 60] using mass spectrometry, chromatography proteolytic digestion, and so forth, on a trial and error basis to find target lysine residues [61]. On larger scale, proteomes having larger and more complex proteins with many SUMOylatable lysine residues are also analyzed through mutational analysis. However, these randomly executed experiments not only take up significant amount of time but also consume physical and chemical toils that are often futile as they result in discovering lysine residues that do not undergo SUMOylation. These approaches focus on identifying substrates rather than exact sites. Computational prediction of target sites has become mandatory before conducting experiments; this enables researchers to directly focus on residues which are potential candidates of SUMOylation. This dry-lab testing prior to corresponding wet-lab experimentation has gained much attention due to its cost effectiveness and power in proteomic data mining. 

Till now, nine prediction models for SUMOylation have been proposed, out of which six have reported servers including SUMOplot [62] (web server), SUMOsp 1.0 and 2.0 [45] (downloadable), SUMOpre [21] (web server), Boshu Liu's PSFS method [63] (web server), SSPFS [51] (available upon request), and seeSUMO [64] (web server).

SUMOplot [62] was the first step in development of computational server for the prediction of SUMOylation sites but had a bias of ΨKXE sites in data. SUMOsp, 2006 [45], was presented as SUMOsp 1.0 and SUMOsp 2.0 that were generated using GPS and MotifX (originally developed for phosphorylation) with sequence information only like SUMOplot [62]. Liu et al. developed a PSFS-based prediction model in 2007 [63] trained on hundreds of amino acid properties from which seven were selected as relevant with the help of sequential forward selection. SUMOpre, 2008 [21], was developed on the basis of sequence data only with probabilistic model of prediction. Its accuracy of 97.7% is impressive but at the cost of its sensitivity which is only 73.96%. SUMOsvm, 2008 [19], was developed using support vector machines trained on sequence information, solvent accessibility, secondary structure, and evolutionary profiles. FindSUMO, 2008 [65], was soon after developed by the PSSM system and with little progress in prediction efficiency. SSPFS, 2009 [51], was developed using mRMR and nearest neighbor algorithm trained on seven optimal amino acid properties selected from hundreds of amino acid properties. SUMOtr, 2010 [23], introduced the use of hydrophobicity, 3D structure, protein volume, and sequence to shape a model through tree classification algorithms. Recently, seeSUMO, 2011 [64], was introduced as a web server using random forest-based algorithm for training, but due to unavailability of its full publication, comparison and information about it have not been included here.

Physicochemical properties including hydrophobicity, buriability, isoelectric point, hydrophilicity, polarity, bulkiness, and molecular weight of residues control the spatial flexibility of target residue and hence can be very important in site attachment by developing complementarity between SUMO, enzymes in the pathway, and the target itself. Here, a computational system (S4) for the prediction of SUMOylation and investigation of its dependence on proposed properties (Table 1) has been developed using random forest-based classifier provided in WEKA [52]. Different programs developed for SUMOylation prediction are not directly comparable as they were developed using not only different datasets but also varied cross-validations and methods. Hence, the MCC, which is designed to assess predictive values for models from classes of different sizes, should be considered the primary measure for the purpose. It has a value between +1 and −1, with +1 being the highest level of prediction, 0 being the average level, and −1 showing inverse prediction.  Table 4 shows the reported AC, SN, SP, MCC, and AUC with their proposed training and evaluation procedures.

In comparison to previous prediction methods that employed highly unbalanced datasets, equivalent amounts of modified and unmodified sites were used to train our model. Previous research (Table 4) has used uneven data with a very large part of the whole dataset being based on unmodified sites. Large number of unmodified instances yields a high specificity by making the correct prediction of nearly all such sites possible, at the cost of lowered sensitivity. Unbalanced dataset reduces performance and reliability so we have made a balanced set by sampling modified and unmodified sites in equal ratios. Moreover, approximately 24% of validated SUMOylation sites do not conform to the consensus motif and their representative peptides have been used in training of this model, and hence, the specificity of our prediction model is for both consensus and nonconsensus sequences, reducing the difficulty faced in prediction of the latter ones. These considerations ensure a uniformity of predictability when the predictor comes across either known or unknown sequences.

Prediction accuracy achieved in this research is significantly higher than all other prediction methods except that of SUMOpre [21] and SUMOsvm [19], to which it is fairly equal. However, our prediction model has exceeded all predictors with its high MCC, sensitivity, and AUC. Particularly, the sensitivity (correct classification of SUMOylated samples) and MCC (the measure of the overall performance of biased datasets) are higher than all others. On specificity (correct classification of non-SUMOylated samples) measures it retains a similar position even after the usage of an equivalent amount of modified and unmodified sites for training.

Based on the prediction performance, we believe that SUMOhunt can very well be used to implement a prediction server in the future that can assist as powerful and complementary tool for SUMOylation site identification and that the model will be available for the purpose. Correct analysis of this modification in all proteomes can greatly enhance our knowledge of the mechanism and working of many biological systems.

## 5. Conclusion

Generating reliable tools for identification of target sites of SUMOylation presents a great challenge. Computational methods of estimation can never replace experimental methods but can be of invaluable support to quicken and focus experimentation. In this research, based on experimental data, a prediction model has been developed that assures of robust computational method for highly accurate and specific SUMOylation-site prediction. Moreover, the physicochemical properties proposed to be playing crucial role in the appropriate presentation and hence rapid SUMOylation of target lysine have brought significant improvement in accuracy measures. This opens new paths to future work in analyzing the effect of these amino acid properties experimentally. Most importantly, based on the model presented here, it gives the possibility of building a server for prediction of SUMOylation sites in relation to the spatial properties of amino acid around them and sequence information.

## Figures and Tables

**Table 1:** Physicochemical properties used to shape SUMOhunt. They were chosen because of their potential contribution in docking of SUMO with substrate protein at the target site.

Amino acid property	AAIndex	Year reported
Hydrophobicity	PRAM900101	1990
Polarity	GRAR740102	1974
Bulkiness	ZIMJ680102	1968
Hydropathy index	KYTJ820101	1982
Accessible surface area	RADA880106	1988
Residue accessible surface area in tripeptide	CHOC760101	1976
Percentage of buried residues	JANJ780102	1978
Entropy of formation	HUTJ700103	1970
Side-chain volume	KRIW790103	1979
Side-chain's contribution to stability	TAKK010101	2001
Buriability	ZHOH040103	2004
Hydrophilicity value	HOPT810101	1981
Molecular weight	FASG760101	1976
Transfer free energy to surface	BULH740101	1974
Steric parameter	CHAM810101	1981
Isoelectric point	ZIMJ680104	1968

**Table 2:** Frequency analysis (occurrence in numbers) of all amino acid residues around modified target lysine in experimentally proved positive dataset of 293 instances.

Residue	−10	−9	−8	−7	−6	−5	−4	−3	−2	−1	0	+1	+2	+3	+4	+5	+6	+7	+8	+9	+10
Ala: A	7	23	24	21	19	17	28	21	24	14	0	13	2	13	9	17	17	18	15	13	18
Arg: R	7	16	10	10	11	16	16	14	13	4	0	10	2	13	9	6	9	7	21	14	16
Asn: N	14	11	9	13	13	13	12	8	12	0	0	6	1	14	9	12	5	12	14	8	5
Asp: D	13	18	16	18	10	11	14	13	16	1	0	10	13	17	32	45	20	24	13	23	13
Cys: C	6	2	8	2	8	2	1	2	7	2	0	3	1	1	2	1	7	3	4	5	11
Glu: E	26	21	21	27	21	28	20	13	23	10	0	33	224	18	34	24	31	37	39	26	19
Gln: Q	10	13	13	17	22	15	19	12	21	3	0	43	2	13	13	13	8	13	12	17	20
Gly: G	17	17	17	14	14	16	14	15	22	4	0	4	6	18	14	12	14	12	12	13	12
His: H	11	8	10	5	7	5	7	7	3	1	0	4	1	4	6	6	6	7	10	8	6
Ile: I	11	14	8	2	19	10	9	14	11	92	0	8	3	9	12	6	6	11	10	9	10
Leu: L	28	22	24	30	18	30	29	22	24	45	0	20	5	22	26	26	18	22	28	21	27
Lys: K	24	19	12	21	21	20	19	32	31	10	293	21	9	25	28	15	20	16	10	22	16
Met: M	14	5	11	7	3	4	10	5	4	6	0	14	2	6	7	7	8	8	3	4	2
Phe: F	8	4	7	12	10	8	6	6	6	9	0	6	5	9	4	13	12	9	7	7	6
Pro: P	26	18	30	33	29	29	27	33	14	6	0	14	7	54	24	17	40	23	15	22	23
Ser: S	23	30	21	28	28	29	29	29	24	6	0	21	4	20	21	37	25	30	27	30	34
Thr: T	18	16	15	11	16	21	17	15	14	5	0	32	3	11	14	14	20	10	10	9	14
Trp: W	3	2	4	2	0	0	1	3	1	0	0	3	0	1	0	4	1	3	0	2	2
Tyr: Y	3	8	7	4	3	4	6	2	6	2	0	4	1	7	8	6	8	7	13	12	6
Val: V	14	20	22	13	19	14	9	27	17	73	0	24	2	17	18	8	10	11	17	15	14

**Table 3:** Accuracy measures on 21-mer, 11-mer, and 7-mer peptides containing target lysine with different combinations of data divided into training and test sets.

% age split	+ve train	−ve train	AC%	SN%	SP%	MCC	AUC	+ve/−ve in test
*21 window size *								
On train	293	291	100	100	100	1.0	1.0	293/291
66%	182	203	82.41	81	84	0.67	0.89	111/88
80%	233	234	88.03	95	80	0.76	0.92	60/57
90%	263	263	89.65	96	82	0.79	0.94	30/28
92%	268	269	93.61	96	90	0.87	0.98	25/22
94%	271	278	94.28	95	92	0.87	0.99	22/13
93%	269	274	87.80	85	93	0.75	0.97	24/17
*11 window size *								
On train	293	291	99.82	99	100	0.99	1.0	293/291
66%	192	193	85.71	87	83	0.70	0.91	101/98
87%	259	249	86.84	97	78	0.75	0.89	34/42
90%	260	266	87.93	90	84	0.75	0.96	33/25
92%	266	271	89.36	88	90	0.78	0.96	27/20
93%	270	273	92.68	95	88	0.85	0.98	23/18
94%	271	278	94.28	95	92	0.87	0.99	22/13
*7 window size *								
On train	293	291	100	100	100	1.0	1.0	293/291
66%	193	192	84.92	87	82	0.89	0.92	100/99
80%	231	236	86.32	91	80	0.72	0.92	62/55
90%	262	264	84.48	90	77	0.67	0.93	31/27
92%	269	268	91.48	96	87	0.83	0.97	24/23
**93%**	**270**	**273**	**97.56 **	**100**	**94**	**0.95**	**1.0**	**23/18**
94%	271	278	97.1429	100	93	0.94	0.99	22/13

**Table 4:** Comparison of SUMOhunt with previous models/servers.

Predictor	AC	SN	SP	MCC	AUC	Training features	Evaluation process	+train/−train
SUMOplot	90%	80%	93%	0.48	—	Only sequence	Training	—/—
SUMOsp (2006) Thd: 18 and Thd: 4	92.71% 80.43%	83.6% 89.12%	93.08% 80.07%	0.5012 0.3232	0.73 —	Only sequence	5-fold and LOOCV	239+ve/—
Boshu Liu et al. (2007)	89.18%	—	—	—	—	Sequence + physicochemical properties	LOOCV	227+ve/226−ve
SUMOpre (2008)	97.71%	73.96%	97.67%	0.6364	0.87	Only sequence	5-fold CV	240+ve/6361−ve Test: 28
SUMOsvm (2008)	97%	62%	99%	0.67	0.92	Sequence + solvent accessibility + secondary structure + evolutionary profiles	5-fold CV	241+ve/ 5741−ve Test: 27+ve/—
SSPFS (2009)	84.4%	—	—	—	—	Sequence + physicochemical properties	LOOCV	191+ve/954−ve Test: 21+ve/106−ve
SUMOtr (2010)	85%	95%	75%	0.68	0.85	Sequence + 3D structure + hydrophobicity	5-fold CV	57+ve/711−ve
SUMOhunt	97.56%	100	94	0.95	1	Sequence + several physicochemical properties	Training + test 10-fold CV	270+ve/273−ve Test: 23+ve/18−ve

## Abbreviations

SUMO: Small ubiquitin-like modifier
NDSM: Negative-charge dependent SUMOylation motif
PDSM: Phosphorylation dependent SUMOylation motif
WEKA: Waikato environment of knowledge analysis
ROC: Receiver operator characteristic
LOOCV: Leave-one-out cross-validation
AC: Accuracy
SN: Sensitivity
SP: Specificity
MAPRes: Mining associationpatterns between preferred residues
MCC: Matthews correlation coefficient
AUC: Area under the curve
PSFS: Properties sequence forward selection
SSPFS: Sumoylation site prediction based on forward selection
PSSM: Position specific scoring matrix
mRMR: Minimum redundancy maximum relevance
dbPTM: Database of post translational modifications
CSV: Comma separated value format
ARFF: Attribute related file.
